# Fear Expression Suppresses Medial Prefrontal Cortical Firing in Rats

**DOI:** 10.1371/journal.pone.0165256

**Published:** 2016-10-24

**Authors:** Thomas F. Giustino, Paul J. Fitzgerald, Stephen Maren

**Affiliations:** Department of Psychology and Institute for Neuroscience, Texas A&M University, College Station, Texas; Creighton University, UNITED STATES

## Abstract

The medial prefrontal cortex (mPFC) plays a crucial role in emotional learning and memory in rodents and humans. While many studies suggest a differential role for the prelimbic (PL) and infralimbic (IL) subdivisions of mPFC, few have considered the relationship between neural activity in these two brain regions recorded simultaneously in behaving animals. Importantly, how concurrent PL and IL activity relate to conditioned freezing behavior is largely unknown. Here we used single-unit recordings targeting PL and IL in awake, behaving rats during the acquisition and expression of conditioned fear. On Day 1, rats received either signaled or unsignaled footshocks in the recording chamber; an auditory conditioned stimulus (CS) preceded signaled footshocks. Twenty-four hours later, animals were returned to the recording chamber (modified to create a novel context) where they received 5 CS-alone trials. After fear conditioning, both signaled and unsignaled rats exhibited high levels of post-shock freezing that was associated with an enduring suppression of mPFC spontaneous firing, particularly in the IL of signaled rats. Twenty-four hours later, CS presentation produced differential conditioned freezing in signaled and unsignaled rats: freezing increased in rats that had received signaled shocks, but decreased in animals in the unsignaled condition (i.e., external inhibition). This group difference in CS-evoked freezing was mirrored in the spontaneous firing rate of neurons in both PL and IL. Interestingly, differences in PL and IL firing rate highly correlated with freezing levels. In other words, in the signaled group IL spontaneous rates were suppressed relative to PL, perhaps limiting IL-mediated suppression of fear and allowing PL activity to dominate performance, resulting in high levels of freezing. This was not observed in the unsignaled group, which exhibited low freezing. These data reveal that the activity of mPFC neurons is modulated by both associative and nonassociative stimuli that regulate conditioned fear.

## Introduction

The medial prefrontal cortex (mPFC) has been shown to play an integral role in learning and memory processes related to emotionally prominent stimuli in both rodents and humans [1–6]. For example, after the conditioning and extinction of fear, the prelimbic (PL) and infralimbic (IL) subdivisions of the mPFC are believed to regulate the expression and suppression of fear, respectively [7–12]. In support of this functional dichotomy, PL inactivation reduces both cued and contextual fear [9], whereas IL inactivation impairs extinction learning [11–15]. Moreover, electrical stimulation of PL enhances fear expression [16], whereas IL activation during extinction enhances extinction recall [17–19]. In addition, inhibitory interneurons in PL coordinate fear expression [20,21], and CS-evoked firing rates in PL correlate with ongoing conditional freezing behavior [10]. In contrast, IL single-neuron CS-evoked responses correlate with extinction recall [7], and the expression of extinction is associated with increased Fos expression in IL relative to PL, a pattern that is reversed during the renewal of fear outside the extinction context (i.e., PL>IL) [22,23]. Reciprocal PL and IL activity during fear expression appears to be mediated in part by ascending basolateral amygdala inputs that themselves show differential activity during low- and high-fear states [24–26].

Although these studies reveal different roles for PL and IL in the regulation of conditional fear, they have largely considered IL and PL in isolation and have focused on CS-evoked spike firing. We have recently observed that fear conditioning is associated with marked changes in the spontaneous firing rate of IL and PL neurons [27] and that reciprocal changes in IL and PL firing appear to correlate with the expression of conditional freezing [28,29]. Here we further explore the relationship between spontaneous and CS-evoked IL and PL firing rates during the acquisition and expression of conditioned freezing in rats. Rats received either signaled or unsignaled footshock and were later presented with the auditory cue in a novel context; simultaneous IL and PL recordings were made during both the conditioning and retention sessions. We find that the expression of both post-shock freezing during conditioning and CS-evoked freezing during retention testing are associated with a differential modulation (largely suppression) of spontaneous firing in IL and PL. This was most pronounced for rats receiving signaled footshocks. In other words, the auditory CS differentially biased spontaneous firing in the mPFC, more strongly attenuating firing in IL relative to PL. We propose that this limits IL-mediated inhibition of fear and allows PL-mediated excitation of fear to dominate performance.

## Materials and Methods

### Ethics Statement

All procedures were conducted at Texas A&M University and were performed in strict accordance with the guidelines and regulations set forth by the National Institutes of Health and Texas A&M University with full approval from its Animal Care and Use Committee (Protocol number: 2015–0005).

### Subjects

Twelve experimentally naïve adult male Long-Evans Blue Spruce rats (weighing 200–224 g; 50–57 days old) were obtained from a commercial supplier (Harlan Sprague-Dawley, Indianapolis, IN). Upon arrival and throughout the experiments, rats were individually housed in cages within a temperature- and humidity-controlled vivarium, and kept on a 14:10 hr light/dark cycle (lights on at 7 am) with ad libitum access to food and water. All experiments took place in the daytime during the light phase. Rats were handled for ~30 seconds a day for 5 days to habituate them to the experimenter before any behavioral testing or surgical procedures were carried out. No animals became ill or died prior to the experimental endpoint.

### *In vivo* electrophysiology

Rats were randomly assigned to one of two groups: 6 rats received “signaled” footshocks during fear conditioning, and 6 others received “unsignaled” footshocks (see below). Prior to *in vivo* electrophysiology experiments, all rats were surgically implanted with a microelectrode array within the medial prefrontal cortex (mPFC). To do so, rats were anesthetized with isoflurane (5% induction, 2% maintenance) and secured in a stereotaxic apparatus (Kopf Instruments, Tujunga, CA). The scalp was incised and retracted; three burr holes were drilled for anchor screws. A portion of the skull overlying mPFC was removed to allow for microelectrode implantation. The rat was implanted with a 16-channel microelectrode array (Innovative Neurophysiology, Durham, NC) targeting both the prelimbic (PL; 8 wires) and infralimbic (IL; 8 wires) subdivisions of the mPFC in the right hemisphere. The 2x8 wire microarray was constructed from two rows of 50 μm diameter tungsten wires of two different lengths (PL, 6.9 mm; IL, 8.0 mm; see below for dorsal-ventral coordinates); wires in each row and the rows themselves were spaced 200 μm apart (center-to-center). The array was positioned with its long axis parallel to the anterior-posterior plane. The coordinates for the centermost wires of the array were (relative to bregma at skull surface): +2.7 mm AP, +0.55 mm ML, -4.0 mm DV for PL; and +2.7 mm AP, +0.35 mm ML, -5.1 mm DV for IL. The array was secured to the skull with dental acrylic and one week was allowed for recovery before *in vivo* recordings began.

A standard rodent conditioning chamber (30x24x21 cm, Med Associates, St. Albans, VT) housed in a sound-attenuating cabinet was modified to allow for electrophysiological recordings. The chamber consisted of two aluminum sides, a Plexiglas rear wall, and a hinged Plexiglas door. The grid floor contained 19 stainless steel rods (4 mm diameter) spaced 1.5 cm apart (center-to-center). Rods were connected to a shock source and solid-state grid scrambler (Med Associates) for the delivery of footshocks. A loudspeaker mounted on the outside of a grating in one aluminum wall was used to play auditory tones. Locomotor activity was transduced by a load-cell under the floor of the chamber, and the output of the load-cell was recorded by an OmniPlex recording system (Plexon, Dallas, TX). All behavioral and neural activity was recorded automatically with this system.

Single-unit recordings occurred over two days in two distinct contexts within this conditioning chamber. On Day 1, the rats were transported to the recording room in a black plastic box, connected to a headstage with a flexible cable, and placed in the recording chamber. The chamber had been cleaned with 1% ammonium hydroxide to provide a distinct olfactory cue, a black pan containing a thin layer of the same solution had been placed under the grid floor, and the room was illuminated with ambient red lights (context A). After a 3-min stimulus-free baseline recording period, “signaled” rats received 5 auditory tone-footshock pairings, whereas “unsignaled” rats received 5 shocks without tones. The tones (conditioned stimuli, CS) were 2 sec, 80 dB, 2 kHz; the shocks (unconditioned stimuli, US) were 0.5 sec and 1 mA, where shock onset occurred at tone offset. There was a 1-min inter-trial interval (ITI) between shocks. Behavioral and neural data were not recorded during this conditioning period due to the electrical noise associated with shock delivery; recordings commenced immediately after the last footshock. The recording session continued for 15 min after the last shock, after which the rat was returned to its home cage. On Day 2, the transport and recording contexts were altered to reduce generalization of fear from the conditioning session to this fear recall test session. The rat was transported in a white plastic box. The recording chamber was cleaned with 1% acetic acid to provide a distinct olfactory cue, a white pan containing a thin layer of the same solution was placed under the grid floor, the grid floor was covered with a transparent rubber mat, the back wall was covered with alternating black and white stripes, and the room was illuminated with ambient fluorescent white lights (context B). After a 3-min stimulus-free baseline period, all rats were presented with five tone-alone trials (1-min ITI); the rat remained in the chamber for 5 min after the final tone and behavioral and neural data were recorded throughout the session. Two rats were excluded from each group for Day 1 and 2 analyses on the basis of being statistical outliers in terms of freezing behavior (1 from each group) or neural activity (1 from each group) (> 2 SD), resulting in group sizes of 4 for signaled and unsignaled.

Extracellular single-unit activity was recorded using a multichannel neurophysiological recording system (OmniPlex, Plexon, Dallas, TX). Wideband signals recorded on each channel were referenced to one of the recording wires (resulting in a maximum of 15 channels of activity per rat), amplified (8,000x), digitized (40 kHz sampling rate), and saved on a PC for offline sorting and analysis. The recording reference wire was located in PL, and was randomly selected to optimize the quality of the recordings. After high-pass filtering the signal at 600 Hz, waveforms were sorted manually using 2-dimensional principal component analysis (Offline Sorter, Plexon). Only well-isolated units were used in the analysis. If two units with similar waveforms and identical time stamps for their action potentials appeared on adjacent electrodes, only one unit was used. Sorted waveforms and their timestamps were then imported to NeuroExplorer (Nex Technologies, Madison, AL) for further analysis.

The analysis of neural activity focused on spontaneous single-unit firing during each recording session; CS-evoked activity was also analyzed for Day 2. For both Day 1 and Day 2, the spontaneous firing rate was normalized to the 3-min baseline period prior to tone and/or shock presentations. In addition, in order to avoid treating neurons collected from the same brain region and rat as independent factors, normalized firing rate data were averaged for all cells collected within a brain region from each rat to create a single value for each brain region over time. This analysis was primarily centered around understanding differences in PL and IL firing. IL normalized activity was subtracted from PL normalized activity to create a PL versus IL difference. For analysis of the CS-evoked activity on Day 2, firing rate was binned in 100 msec increments around the time of the tones for individual neurons, and the evoked responses were normalized to the 1 sec period prior to tone onset, averaged across the 5 tones.

### Histology

After the completion of experiments, rats were overdosed with pentobarbital and electrolytic lesions were created by passing electrical current (80 μA, 10 sec; A365 stimulus isolator, World Precision Instruments, Sarasota, FL) through 6 of the recording wires (anterior, middle, posterior wires in both PL and IL) to mark the location of the recording array in mPFC. The rats were then perfused transcardially with 0.9% saline followed by 10% formalin. Brains were extracted from the skull and post-fixed in a 10% formalin solution for 24 hours followed by 10% formalin/30% sucrose solution where they remained for a minimum of 48 hours. After the post-fix period, coronal brain sections (50 μm thickness) were cut on a cryostat (-20° C), mounted on subbed microscope slides, and stained with thionin (0.25%) to visualize electrode placements. Electrodes that were located outside of PL or IL, which principally consisted of those that were too lateral, were excluded from further analysis. We did, however, obtain neurons from both PL and IL in each of the rats.

### Statistics

Data were analyzed with conventional parametric statistics (StatView, SAS Institute). Two-way analysis of variance (ANOVA) and repeated-measures ANOVA were used to assess general main effects and interactions (α = 0.05). Paired and unpaired student’s two-tailed *t*-tests were also used for pairwise comparisons of means. Results are shown as mean ± SEM.

## Results

### Spontaneous mPFC firing and post-shock freezing

**Fig 1:**
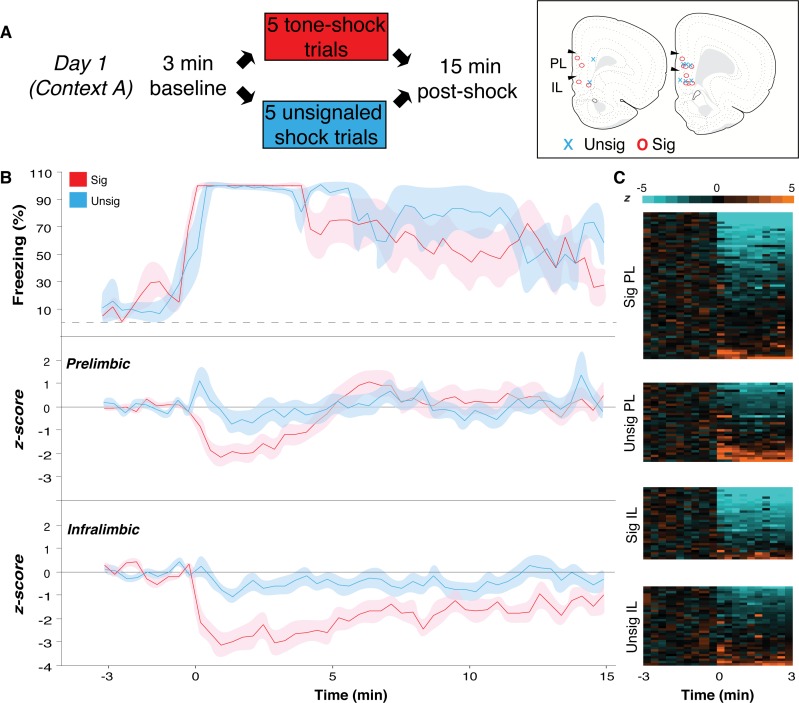
Signaled and unsignaled footshock modulates mPFC single-unit activity. **(A)** Day 1 experimental design; histological placement of the center of each electrode array in mPFC is shown. Each array targeted both PL (8 wires) and IL (8 wires) in the right hemisphere. Coronal sections represent (left to right) coordinates +3.2 and +2.8 relative to bregma in the anteroposterior plane. **(B)** Signaled (red) and unsignaled rats (blue) displayed no significant difference in freezing behavior during the post-shock 15 min stimulus-free period. The spontaneous firing rate of all neurons was normalized to the pre-conditioning baseline for each brain region and shock group. Normalized firing rate for PL and IL for signaled and unsignaled, (time 0 is immediately after the last shock). **(C)** Heat maps showing shock-induced changes in firing rate for individual neurons split by group and brain region. Data during the shock trials were not recorded. All values are means ± SEM.

To examine the effects of signaled and unsignaled footshock on mPFC neural responses, we performed single-unit recordings in freely moving rats. Animals were implanted with a 16 channel microelectrode array in the right hemisphere, targeting both PL (8 wires) and IL (8 wires). Placements of the center of the array for each animal are shown in Fig 1A. Animals were given a one week recovery period following implantation surgery. For behavioral testing, rats were transported to a behavioral chamber which was modified to allow for awake, behaving recordings. A headstage and flexible cable connected the rat to a multichannel Omniplex recording system (Plexon) to allow for the collection of neural data. Freezing behavior was measured automatically using a load-cell transducer and amplifier [30]. Single-units recorded in separate sessions were treated as separate neurons.

In the first recording session (Day 1), after a stimulus-free 3 min baseline period, rats received either 5 tone-footshock trials (“signaled” rats) or 5 unsignaled footshock trials (“unsignaled” rats) and remained in the chamber for 15 min after the last shock was delivered (see Fig 1A for Day 1 design). The recording chamber had specific visual and olfactory cues to make the context distinct (see Materials and Methods). Neural and behavioral data were recorded throughout the session, except during shock delivery and the inter-shock intervals due to electrical noise caused by coupling the grid floor with our recording system.

Freezing and spontaneous firing rate data were averaged in 20-sec bins [Fig 1B and 1C; time 0 is immediately after the last shock]. A repeated measures ANOVA revealed no difference in post-shock freezing between shock groups [Fig 1B; main effect of group, *ns*; group x time, also *ns*]. However, both signaled and unsignaled shock significantly modified the spontaneous firing rate of PL and IL neurons, consistent with a previous report from our laboratory (27) (Fig 1B and 1C). Heat maps display shock-induced changes in firing rate for each neuron split by group and brain region (signaled PL n = 63, unsignaled PL n = 34; signaled IL n = 31, unsignaled IL n = 34) (Fig 1C). In signaled rats, the populations of PL and IL neurons showed a pronounced suppression of spontaneous firing rate in the immediate post-shock period, which was greater in magnitude and longer lasting in IL. In unsignaled rats, the post-shock changes in PL and IL firing were qualitatively and quantitatively different. In this case, PL neurons showed a transient increase in firing, whereas IL neurons showed a modest suppression of firing rate. An ANOVA performed on these data revealed that these changes in firing rate were dependent on the conditioning procedure, the brain area where the recordings were made, as well as an interaction of these factors [main effect of group, *F*(1,158) = 4.75 *p* < 0.05; main effect of brain region, *F*(1,158) = 6.73, *p* < 0.05; time x group x brain region interaction, *F*(47,7426) = 1.48, *p* < 0.05]. Importantly, spontaneous firing in IL was suppressed much more than PL firing, and this was most robust in the immediate post-shock period, when freezing was highest.

### Spontaneous and evoked mPFC firing during the expression and inhibition of conditioned fear

**Fig 2:**
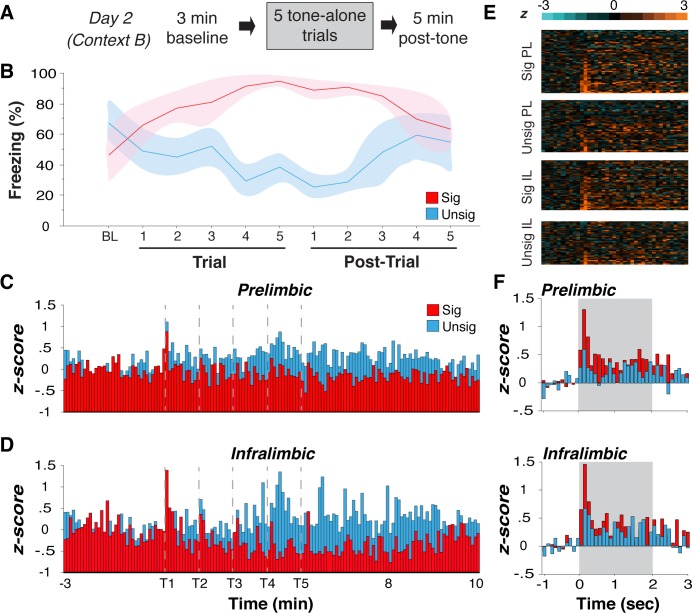
Fear recall suppresses mPFC population level single-unit activity. **(A)** Day 2 design. **(B)** Upon tone presentation on Day 2, signaled (red) and unsignaled rats (blue) displayed opposing freezing patterns. (**C, D**) Average normalized firing rate data from PL and IL are binned in 5 sec increments for the duration of the session. Gray dashed lines indicate tone onset (tone response is the bar immediately to the right of dashed line) for the 5 tones. Signaled rats exhibited robust tone-evoked responses that are superimposed on the overall suppression of neural activity in both PL and IL, as compared to the general increase in firing in unsignaled PL and IL. **(E)** Heat maps plotting tone-evoked responses for neurons from signaled and unsignaled rats. **(F)** Normalized tone-evoked histogram averages plotted in 100 msec bins for PL and IL. Previously signaled rats showed robust tone-evoked responses in both PL and IL around tone onset (first 200 msec) compared to unsignaled rats. No difference was observed between brain regions when comparing within a shock group. All values are means ± SEM.

Twenty-four hours after conditioning, the rats were transported back to the recording chamber for a retention test. The recording context for this session was distinct from that used for conditioning, although there was nonetheless a moderate level of generalized fear that was likely associated with tethering the rats to the recording cable in both sessions. Three minutes after placement in the chamber, the rats received 5 auditory cues (1 min ISI, see Fig 2A for design). This session served to index conditioned fear to the CS in the signaled rats; unsignaled rats were not expected to freeze to the tone. Indeed, as shown in Fig 2B, presentation of the auditory CS increased conditional freezing in signaled rats and decreased freezing in the unsignaled rats [Fig 2B; main effect of group, *F*(1,6) = 9.65, *p* < 0.05; time x group interaction, *F*(10,60) = 3.30, *p* < 0.01]. The decrease in freezing in the unsignaled rats is likely the result of external inhibition of conditional responding by the novel auditory cue [31]. In other words, the novel auditory tone served as a “distracting” stimulus that reduced conditional freezing.

The differential freezing behavior in signaled and unsignaled rats was associated with differences in both spontaneous and CS-evoked firing in PL and IL. As shown in Fig 2C and 2D PL and IL neurons from signaled rats showed robust short-latency excitatory responses to the tone CS (dashed lines). The tone-evoked responses among PL and IL neurons are evident in the heat maps depicting CS-evoked firing from individual neurons split by group and brain region (Fig 2E), which correspond to the average peri-event histograms aligned to tone onset (Fig 2F). These histograms reveal that the excitatory tone-evoked response (averaged across the first 200 msec of tone onset) was significantly greater in neurons from signaled rats compared to those from unsignaled rats for both PL [*t*(102) = 2.46, *p* < 0.05] and IL [*t*(82) = 3.38, *p* <0.01]. The magnitude of the tone-evoked response did not differ between PL and IL within each conditioning group.

Interestingly, however, the spontaneous firing rates of the population of PL and IL neurons did show differential changes in firing rate in the two groups. As shown in Fig 2C and 2D neurons from signaled rats exhibited a pronounced decrease in firing rate across the session, particularly in IL. This is in contrast to neurons from unsignaled rats, which exhibited increases in spontaneous firing rate over the session. An ANOVA performed on these data reveals that the pattern of firing across the session was dependent upon the conditioning procedure [*F*(1,176) = 18.88, *p* < 0.01] and interacted with brain region [time x group x brain region interaction, *F*(146,25696) = 1.20, *p* < 0.05)]. In other words, the expression of conditioned fear to the CS in the signaled rats was associated with a differential decrease in IL and PL firing rates (i.e., IL more suppressed than PL) compared to a similar increase in IL and PL firing rates in unsignaled rats. Importantly, no difference in raw firing rate was observed during the 3 min baseline period prior to CS presentation [main effect of group, *ns*; main effect of brain region, *ns*; group x brain region, *ns*] or the generalized level of baseline freezing [unpaired t-test, *ns*], so it is unlikely that these factors impacted the observed differences.

### Differential firing in PL versus IL predicts freezing behavior

**Fig 3:**
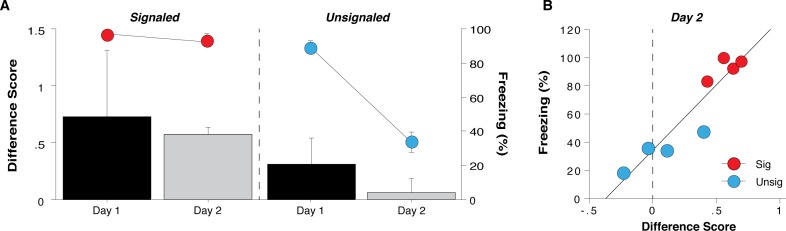
Differential firing in PL versus IL predicts freezing behavior. **(A**) Freezing (circles) was similar across Days 1 (3 min post-shock period) and 2 (tones 4 and 5) in signaled rats, whereas it decreased markedly on Day 2 in unsignaled animals. Relative neural activity (PL minus IL) was similar in magnitude (and positive) across days for the same time periods in signaled rats, but also decreased in magnitude markedly on Day 2 in unsignaled animals. **(B)** Linear regression analysis showing the PL vs IL difference during tones 4 and 5 plotted against freezing levels for the same time period. This analysis revealed a strongly positive correlation between the two variables when including rats from both shock groups. All values are means ± SEM. *p < 0.01.

During both conditioning and retention testing, fear-induced changes in IL and PL spontaneous firing rate were different, particularly in the signaled group. To further examine the relationship between conditioned freezing behavior and mPFC firing rate in each rat, we averaged the normalized firing rate in each brain area for a 3-min period after the final footshock (Day 1) or a 2 min period during tones 4 and 5 (and the ITIs) from the Day 2 test trials. These time points were chosen because both groups exhibited asymptotic levels of freezing during this period on Day 1. In addition, for Day 2 this was the time point of largest separation in freezing between groups. We then generated a difference score by subtracting IL from PL (PL-IL) for each session and rat and plotted these difference scores in relation to the average freezing behavior across the same time periods. As shown in Fig 3A, differences between PL and IL firing were lowest in the unsignaled group during the test session, a group that also showed the lowest conditioned freezing [Day 2 sig vs unsig; *t*(6) = 3.61, *p* < 0.05]. Importantly, no difference was observed between groups on Day 1, when freezing was similarly elevated. In other words, a greater difference between PL and IL firing rates driven by a larger suppression of IL relative to PL firing was associated with high levels of conditional freezing. Interestingly, the difference in normalized PL and IL firing rates strongly predicted freezing behavior during the test session, in particular. Indeed, when freezing was most different between the groups in the later trials of the test (trials 4 and 5), there was a highly significant positive correlation between the PL-IL firing rate differential and freezing behavior (where PL firing in the signaled group was less suppressed than IL firing, resulting in a positive PL-IL difference value) [Fig 3B; Pearson *r* = 0.93, *p* < 0.01]. These data reveal that the balance of spontaneous neural firing in PL and IL is a strong predictor of freezing behavior.

## Discussion

As we have previously reported, footshock stress rapidly and persistently alters neural signaling in both PL and IL [27]. Somewhat unexpectedly, we also found here that the magnitude and direction of these firing rate changes is altered by the presence of an associative cue during the acquisition of conditioned fear (i.e., signaled vs unsignaled shock), where we observed greater post-shock suppression of spontaneous firing in mPFC of signaled rats. We also show here, to our knowledge for the first time, that the mPFC is engaged during the nonassociative external inhibition of fear. In contrast to rats that received signaled footshocks during conditioning, rats receiving unsignaled shock exhibited decreases in freezing upon presentation of the auditory stimulus on Day 2. These opposing behavioral responses were mirrored in the spontaneous firing rates of mPFC neurons. Signaled rats showed a suppression of PL and, to a greater extent, IL activity, whereas unsignaled rats displayed increased PL and IL firing. Interestingly, we also observed that the difference in PL versus IL firing rate strongly predicts freezing behavior upon fear recall: signaled rats showed a relative bias favoring PL over IL activity and this difference was not observed during the external inhibition of fear. Collectively, these data suggest differences in PL and IL signaling may underlie freezing levels, rather than PL activity alone [10]. These data also reveal a general role for the mPFC in fear regulation that extends to both associative and nonassociative learning mechanisms.

A number of studies have revealed that the mPFC has an important role in fear conditioning and extinction [1,2,6,8,32]. It is largely believed that within the fear circuit PL and IL have opposing roles, with PL activity underlying fear expression and IL fear suppression [7–15]. While both the signaled and unsignaled group displayed asymptotic levels of freezing immediately following the last conditioning trial, we observed differential firing rate patterns between groups. Signaled rats showed a general suppression of mPFC firing activity in both PL and to a greater extent IL. This mPFC suppression may be driven by ascending amygdala input [33]. In contrast, we observed a moderate suppression of IL firing in unsignaled rats and a transient peak in firing in PL. It is possible that these findings reflect differences in the acquisition or consolidation of cued (signaled) versus context (unsignaled) fear which may relate to differences in the temporal predictability of the US. For example, the peak in PL firing in the unsignaled group after the last conditioning trial may reflect a potential timing mechanism in anticipation of an upcoming shock.

Several previous studies have examined PL or IL CS-evoked activity upon fear recall, or extinction and retrieval [7,10,20,27,28,28,29]. Yet how concurrent PL and IL activity, and more specifically spontaneous firing rate, relates to ongoing freezing levels has not been addressed. Given that IL activation is thought to underlie the suppression of fear after extinction (a low fear state), it is possible that IL suppression, rather than PL excitation (or a combination of the two), represents a neural mechanism for fear expression. Indeed, we show that whereas PL spontaneous firing is not necessarily enhanced upon fear recall (high fear), IL firing is consistently more suppressed (particularly in signaled rats). This observation is in agreement with previously reported slice recordings which demonstrated that fear conditioning dampens the intrinsic excitability of IL neurons and this can be reversed with extinction training [34,35]. These data suggest that a *suppression* of IL activity may underlie fear expression. It is possible that this represents a relative shift in PL vs IL influence on the fear state, favoring high fear with a larger suppression of IL activity, creating a bias towards PL activity, as observed in the signaled group here. Interestingly, in the unsignaled group, where fear was reduced during tone presentation, PL and IL neural activity were moderately increased and largely indistinguishable between the two regions. Under these conditions, there would be no bias favoring either brain region which may relate to the observed decrement in freezing. Importantly, no difference was observed between groups in terms of generalized baseline freezing or raw firing rate during this session, making it unlikely that these factors contributed to the observed differences between groups upon CS presentation, including what we interpret as external inhibition. These data suggest that a relative shift in PL vs IL signaling, as opposed to the activity of either brain region alone, may be a strong and reliable predictor of freezing behavior. While little is known regarding PL-IL functional connectivity, it is likely that these differences stem from both cortico-cortical connectivity as well as ascending input from the amygdala [24–26] which may differ between groups.

The fact that PL and IL activity were nearly indistinguishable in unsignaled rats (low freezing) on Day 2 suggests a broader role for the mPFC in the nonassociative inhibition of conditional fear. Little is known about the neural substrates underlying nonassociative mechanisms that may reduce conditional fear, such as external inhibition. While a wealth of data exists on nonassociative mechanisms of learning, the findings primarily focus on habituation (decreased responding to repeated presentation of a stimulus) and sensitization (increased responding to repeated stimulation). In addition, these studies often investigated hippocampal mechanisms of nonassociative learning [36–39] and do not directly focus on aversive processes. In terms of conditioned fear, many studies have shown that stress exposure can facilitate Pavlovian conditioning via nonassociative mechanisms (e.g., sensitization) [40–42]. Interestingly, our laboratory has previously shown that the hippocampus mediates the recovery of extinguished fear in response to unexpected events (i.e., external disinhibition) [43]. In this design, after fear extinction, rats were presented with a familiar CS in a novel context or a novel cue in a familiar context. In either case, rats exhibited fear renewal and this could be blocked by hippocampal inactivation [43]. These data point to a key role for the hippocampus in the renewal of fear to an extinguished CS or a novel stimulus, although it remains possible that mPFC activity, through interactions with the hippocampus, underlies this nonassociative relapse of fear as well. Here, we have shown that in animals trained with unsignaled shocks, a novel cue presented at test produces the external inhibition of conditional fear. Interestingly, this corresponded to moderately elevated firing in both PL and IL, suggesting the mPFC may play a broader role in the nonassociative inhibition of fear. Consistent with this idea, a recent study has proposed that presentation of a novel stimulus during extinction learning serves to reduce post-extinction recovery of fear in both rats and humans [44]. It is possible that this novelty-induced facilitation of extinction is mediated by preferentially engaging the mPFC, although this remains an open question.

In summary, here we have extended previous research examining footshock-induced changes in mPFC activity. Additionally, we observed medial prefrontal correlates of external inhibition in which increases in mPFC firing in unsignaled rats were associated with decreased freezing to a novel stimulus, with little to no difference in PL versus IL spontaneous firing. These data reveal a broader role for the mPFC in the regulation of inhibitory processes, extending beyond fear extinction to include the nonassociative external inhibition of conditional fear. In contrast to the findings in the unsignaled group, signaled rats showed a large difference between PL and IL firing during fear recall, which strongly predicted high freezing behavior. Overall, our data suggest that the difference in PL versus IL activity, driven by a suppression of IL, may underlie fear expression, rather than the activity of either brain region alone.
